# Identical Attentional Capture with Different Working Memory Representation Precision

**DOI:** 10.3390/bs16010104

**Published:** 2026-01-13

**Authors:** Liangliang Yi, Ruikang Zhong, Haibo Zhou, Daoqun Ding, Yutong Liu, Xinxin Xiang, Yaru Yang

**Affiliations:** 1 Department of Psychology, School of Education Science, Hunan Normal University, Changsha 410081, China; liang@hunnu.edu.cn; 2 School of Education, Hunan University of Science and Technology, Xiangtan 411201, China; seazx@mail.hnust.edu.cn (R.Z.); hbzhou@hnust.edu.cn (H.Z.); yutongaria@mail.hnust.edu.cn (Y.L.); xinxiang@mail.hnust.edu.cn (X.X.); youyou@mail.hnust.edu.cn (Y.Y.)

**Keywords:** resource allocation, attentional capture, visual search

## Abstract

Attention can be automatically captured by the distractor that matches the representation of working memory (WM) in search tasks, impairing visual search efficiency and resulting in attentional capture effects. The resource hypothesis of visual search predicts that resource allocation affects attentional capture. However, previous studies have shown partly opposing results inconsistent with this prediction. The purpose of this study is to assess the connection between attentional capture and WM resource allocation. Two experiments were conducted to combine the attentional capture paradigm with continuous delayed-estimation tasks. In Experiment 1, we manipulated the number of memory items between one and two and measured the WM representation precision as well as the magnitude of attentional capture. In Experiment 2, we manipulated resource allocation using a retro-cue task with the presentation of two memory items. In Experiment 1, the results show that when remembering one item, a single-item representation had higher precision compared to the scenario for remembering two items, and it also involved a greater allocation of WM resources. However, there was no significant difference in the magnitude of attentional capture effects between the two conditions. In Experiment 2, the results show that memory precision was higher when the cue pointed to the item compared to when it did not, but there was no significant difference in the magnitude of attentional capture effects between the cued-match and non-cued-match conditions. The findings show that the size of attentional capture effects based on WM is unaffected by the distribution of WM resources. Attentional capture effects may reflect the attention bias of WM representation that occurs in preparation stage of memory-based attentional guidance.

## 1. Introduction

The brain’s cognitive processing mechanism, known as visual working memory (VWM), allows us to temporarily store and alter visual information, enabling us to recall three to four visual objects at once (Cowan, 2001; Ma et al., 2014; Park & Zhang, 2024). VWM and visual attention are closely related. It is commonly accepted that VWM keeps a goal template for visual search and permits competition from other things (Bundesen, 1990; Duncan & Humphreys, 1989). As a result, VWM’s template directs attention to objects in the visual scene that are more like the target, improving visual search effectiveness. It has also been demonstrated that objects that are kept in VWM but unrelated to the search objective automatically direct visual attention. According to the biased competition hypothesis, the cerebral cortex encoding properties associated with a representation becomes preactivated when it is stored in VWM. Therefore, if an item matching the working memory (WM) representation reappears in the field of vision, it will guide the visual attention. This representation is known as the attentional template (Desimone & Duncan, 1995; Zivony & Eimer, 2024). Extensive subsequent research has provided further evidence for the bias competition model and has demonstrated that in visual search tasks, attention can be guided by VWM representations (Che et al., 2024; Soto et al., 2005, 2006; Yang et al., 2024). For example, previous studies have combined WM tasks with visual search tasks and have made WM representations share the same features as search targets or distractors with visual search tasks. The results indicate that visual search stimuli sharing features with WM representations, whether as targets or distractors, can obtain more attention bias than other search stimuli that do not share features (Soto et al., 2005, 2006), which demonstrates attentional guidance effects. When the stored WM representation matches the search target, attentional bias enhances search efficiency. Conversely, when the WM representation has the distractor stimulus, attentional bias will impair search efficiency.

Although the link between attention and VWM is well established, the exact nature of this interaction remains under debate. The single-item-template (SIT) hypothesis, inspired by state-based models of working memory (McElree, 2001; Oberauer, 2002), proposes that working memory consists of two representational states: accessory representations, which cannot influence visual search until they become relevant (Olivers et al., 2011), and a single “active” representation maintained through attentional focus. In contrast, the multiple-item-template (MIT) theory argues that several representations can guide visual search simultaneously (Beck et al., 2012), through a broader focus of attention (Bahle et al., 2020), and can maintain a small set of representations (Gilchrist & Cowan, 2011). Both hypotheses are supported by corresponding empirical evidence. In recent years, an increasing number of studies producing behavioral results (Bahle et al., 2020; Irons et al., 2012; Moore & Weissman, 2010; Roper & Vecera, 2012; Williams et al., 2023), electrophysiological evidence (Bahle et al., 2020; Berggren et al., 2020; Christie et al., 2015; Grubert & Eimer, 2015, 2016; Huynh Cong & Kerzel, 2021; Irons et al., 2012; Moore & Weissman, 2010; Roper & Vecera, 2012) and eye-tracking evidence have found that two WM representations can simultaneously guide visual search (Beck & Hollingworth, 2017; Beck et al., 2012), supporting the MIT hypothesis.

The recently proposed resource models of WM provide new insights into how WM interacts with attention (Huynh Cong & Kerzel, 2021). As a limited resource, WM allows individuals to flexibly and strategically allocate resources among representations. WM resources can reduce interference from sensory stimuli and enhance the precision of WM representations (Bays et al., 2009; Kerzel & Witzel, 2019; Ma et al., 2014; W. Zhang & Luck, 2011). The results of recent studies demonstrate that the relationship between WM representations and visual search is also influenced by the distribution of WM resources (Huynh Cong & Kerzel, 2021). For example, Kerzel & Witzel employed the contingent attentional capture paradigm and instructed participants to memorize a search target and a distractor item with equal precision before the search task began (Kerzel & Witzel, 2019). It was found that only the target item captured attention, establishing an attentional template, while the distractor item did not capture attention.

To better understand the link between resource allocation and visual search, Huynh Cong and Kerzel proposed three ideas (Huynh Cong & Kerzel, 2021): first, forming a representation capable of guiding visual search requires more than simply assigning it the maximum amount of WM resources (Dube & Al-Aidroos, 2019; Dube et al., 2019). Second, the amount of WM resources allocated to representation is proportional to its relevance for visual search. Third, the effectiveness of a representation in guiding visual search depends on the resources devoted to it, whether by strengthening visual attentional guidance, facilitating target detection, or ensuring that representation remains stable and undisturbed. Subsequent research employing the dual-target search paradigm found that SIT search conditions have higher search efficiency and allocation of WM resources than dual-item-template search conditions. In dual-item-template search conditions, templates with higher resource allocation exhibit greater search efficiency compared to those with lower resource allocation. Moreover, templates with higher search relevance consistently receive more of the allocation of WM resources, demonstrating the impact of the allocation of WM resources on attentional guidance (Huynh Cong & Kerzel, 2022).

With the attentional capture paradigm, however, the connection between the distribution of WM resources and attentional guidance has received little attention. Within this paradigm, it is necessary to suppress the attraction to the matching distractors if WM representations match the distractors of the search task. According to the biased competition model, participants tend to allocate attention toward distractors that are congruent with memory items, which reduces the search efficiency (Desimone & Duncan, 1995). Therefore, compared to non-matching distractors, matched distractors capture more attention and reflect an attentional capture effect based on WM representations. Compared with the dual target search paradigm, there are still difficulties and controversies in using the attentional capture paradigm to study the relationship between WM resource allocation and attention guidance. First, the attentional capture effect is not consistently stable. Some studies have found that distractors matching WM representations do not attract attention (Arita et al., 2012; Wen et al., 2018; Woodman & Luck, 2007). However, other researchers have observed that distractors matching WM representations can capture attention (Hollingworth & Beck, 2016; van Moorselaar et al., 2014). In terms of the number of memory items, some studies have found that only one WM item can capture attention (Hollingworth & Beck, 2016; van Moorselaar et al., 2014), whereas other researchers have found that multiple WM representations can simultaneously capture attention (Che et al., 2021). The inconsistent results may be related to different search tasks, WM precision requirements, and distinct types of WM representations (Che et al., 2021; Fan et al., 2019). Subsequent studies have found that differences in attentional resource consumption related to task difficulty and WM resource consumption are two important factors contributing to the variation in the inhibition of distractor effects (Emrich et al., 2010). This is an important reason for the difference in the inhibitory effect of distractors (Dube et al., 2016; Wen et al., 2018; Che et al., 2021).

The resource theory of visual search, which predicts how WM resources will affect attention capture effects, is also controversial because it contradicts several research findings. According to the resource hypothesis of visual search (Huynh Cong & Kerzel, 2021), the precision of WM representations directly indicates the allocation of resources among stored representations, with high-precision WM representations increasing attentional bias toward relevant features. In the attentional capture paradigm, the allocation of WM resources to a representation should correlate directly with its attentional capture effect. For instance, a single WM representation with a memory-set-size 1 should receive more WM resource allocation than a representation with a memory-set-size 2. However, multiple studies have found that the presence of a distractor item that matches the memory item does not yield a significant difference in attention capture between remembering one item (load 1) and two items (load 1) (Fan et al., 2023; Hollingworth & Beck, 2016; Che et al., 2021; Fan et al., 2019). This result contradicts the hypothesis and suggests that WM resources cannot predict attentional guidance (Huynh Cong & Kerzel, 2022). In addition, the WM detection tasks in the aforementioned studies all used change detection procedures. This paradigm focuses more on measuring the accuracy of memory, but it is difficult to effectively measure the precision of WM representations. In continuous delayed-estimation tasks, the gap between an item’s actual features and the features reported on each trial can reveal both the accuracy of item detection and how resources are distributed across representations, offering an alternative to previous measurement approaches (Fougnie et al., 2012; Rademaker et al., 2012; Wilken & Ma, 2004; Zokaei et al., 2011). Attentional capture denotes the increase in response time when the search display contains an item matching working-memory content, operationalized as the RT difference (RT_{match} − RT_{non-match}). Attentional guidance refers to the mechanism by which working memory biases selection priorities; capture is one observable consequence of such guidance but does not exhaust it. Visual search efficiency denotes the rate at which response time scales with set size (ms/item), typically indexed by the slope of the RT–set size function, and should not be equated with capture magnitude.

The purpose of this study was to learn more about the relationship between attentional capture and WM resource allocation. An attentional capture paradigm was used to assess the capture magnitude. Continuous delayed-estimation tasks were employed to measure the precision of WM representations, and the allocation of resources between different WM representations was adjusted by manipulating retro-cue and changing the amount of WM items. The results of experimental tasks show that continuous delayed-estimation tasks could enhance the precision requirement of WM representations, leading to higher WM resource consumption (W. Zhang & Luck, 2011), reduced cognitive control resources, and decreased inhibition of distractors (Che et al., 2021). Additionally, the gap-location task in search could reduce search efficiency and aid in investigating the attentional capture effects. This study consisted of two experiments. Experiment 1 involved one or two WM items, controlled the number of matching distractors in the search task, and used continuous delayed-estimation tasks to analyze the difference in attentional capture and WM representation resource allocation for different WM representations. On the basis of Experiment 1, Experiment 2 presented participants with two WM items and manipulated resource allocation between them using the retro-cue paradigm to measure the difference in attentional capture of WM representations under different WM resource allocations.

## 2. Experiment 1

### 2.1. Material and Methods

#### 2.1.1. Participants

Thirty-two undergraduate students (aged 19–22 years, M = 20.13) from Hunan University of Science and Technology, who were not psychology majors, were enrolled in the study. Among them were fifteen males and seventeen females. After the experiment, all participants received a payment of RMB 30 (~USD 4.15). Every subject gave their informed consent and stated that they were right-handed, had normal or corrected vision, and were not color blind. The Ethics Committee of Hunan University of Science and Technology’s School of Education examined and approved this work in accordance with the Declaration of Helsinki (2024-03).

#### 2.1.2. Stimuli and Apparatus

The experiment was adopted from previous research (Dube & Al-Aidroos, 2019; Hollingworth & Beck, 2016; Huynh Cong & Kerzel, 2022; B. Zhang et al., 2018; Zhou et al., 2020) with Psychtoolbox-3 on MATLAB 2019B (Brainard, 1997; Pelli, 1997). The experiment’s colors were defined using the CIELAB color space, a color-appearance model in which perceived differences are represented by distances within the space. All stimuli were presented against a gray background with a luminance of 15.5 cd/m^2^. The color wheel consisted of 180 values evenly distributed throughout the CIELAB space, forming a circle with a radius of 60 and centered at (L = 70, a = 20, b = 38). For each trial, colors for the memory items and color distractors in the search task were randomly selected from the wheel, with the constraint that any two chosen colors be separated by at least 50° on the 360° wheel.

One- or two-colored squares (1.32° × 1.32°) made up the memory array; the colors were chosen at random from the color wheel. The memory elements were shown at random points in a 3.66° radius virtual circle that was centered at the central fixation point. If two memory items were presented, they were positioned 180° apart from the center. The visual search array comprised eight outlined squares with a gap on the top, bottom, left, or right (0.88° × 0.88°, line thickness 0.15°, referred to as squares below), evenly distributed on a virtual circle centered at the screen’s central fixation point with a radius of 3.66°. In each search task, the target square had a gap on the left or right, and seven of the squares had a gap on the top or bottom. Among the eight squares, either zero (all eight squares were white, serving as the baseline) or two-colored squares were randomly presented as distractors. The colored distractors were not targets for the search task, and their colors could either match or not match the colors of the memory items. In the memory test task, a colored wheel was presented with its center at the central fixation point and a radius of 8.2°, with a thickness of 2.2°. Between trials, the zero-hue angle’s spatial orientation was changed at random. At the location of the original memory item, a black square with no fill color inside the square emerged within the colored wheel. If there were two original memory items, one of the two squares was probed (highlighted by a thick black line). If there was only one original memory item, a square appeared at its original position.

In Experiment 1, each trial began with a “+” displayed at the center of the screen for 500 milliseconds. This was followed by a memory array, presented for 300 ms, containing either one or two items. Participants were later tested on their memory for both the locations and colors of these items. In the memory test, they selected the color on the color wheel that most closely matched the probed memory item. After a 500 ms delay, the trial proceeded in one of two ways: in the visual search task, participants located the target and indicated the position of its gap.

In Experiment 2, the memory array always contained two items. Unlike in Experiment 1, the delay phase following the memory display was replaced with a 200 ms retro-cue. This cue correctly indicated the item to be tested in the subsequent memory task on 75% of the trials. Additionally, in the visual search task, colored squares appeared either once or not at all.

#### 2.1.3. Procedure

**Figure 1:**
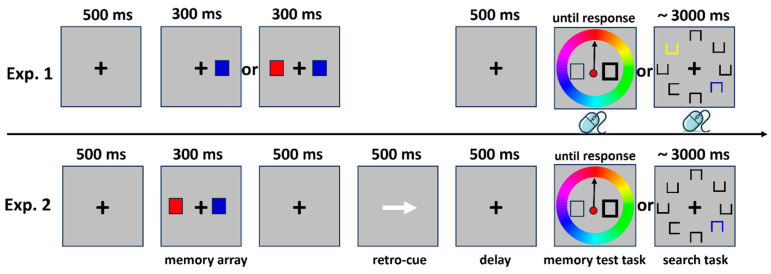
Trial sequence.

In Figure 1, an example trial is shown. The participants were seated about 65 cm away from the computer screen. A “+” was displayed in the middle of the screen for 500 milliseconds at the start of each trial. Subsequently, a memory array was presented, which consisted of either one or two items, with a presentation time of 300 ms. Participants were required to remember the positions and colors of these items to complete subsequent memory test tasks. After a 500 ms delay, the trial ended in one of two ways: in the memory test task, the location of the original memory item on the color wheel was indicated by a black square outline with no color fill. The bold outline indicated which square corresponded to the memory item that needed to be recalled. Participants were required to move an arrow with a mouse to select the color on the color wheel closest to the probed memory item. In the visual search task, eight outlined squares (each 0.44° in width and height) were arranged in a circle around the fixation point. The target square had a gap on either the left or right side, while the other seven squares had gaps on the top or bottom. Participants were instructed to locate the target and indicate the gap position using their right hand to click. Each participant completed 180 memory trials and 180 search trials (30 trials for each of the six matching conditions), totaling 360 experimental trials. Before the main experiment, they completed 10 practice trials—five for the memory test task and five for the search task—to familiarize themselves with the procedures. Rest breaks were provided during the experiment.

#### 2.1.4. Design

This experiment employed a 2 (number of memory items: 1, 2) × 4 (matching conditions: baseline, match 0, match 1, match 2) repeated measures design. The experimental conditions are outlined below. The number of memory items refers to the number of colored squares that the participants were required to memorize in the WM test task. The matching conditions refer to the relationship between the colors of the memory items and the colored distractors in the visual search task (matching indicates that the color of the subsequent search task’s distractor corresponds with that of the preceding memory item). The baseline condition refers to a scenario in which, irrespective of the colors presented during the preceding memory array, all target and distractor stimuli in the visual search task are white squares. The match 0 condition indicates the presence of two-colored distractors in the search task, with colors that do not match those of the preceding memory items. Match 1 condition indicates that one of two colored distractors matches the color of the preceding memory item in the search items. Under match 2 condition, both colored distractors in the search task match the color of the preceding memory item. There is a total of seven combinations of conditions (memory 1—baseline; memory 1—match 0; memory 1—match 1; memory 2—baseline; memory 2—match 0; memory 2—match 1; memory 2—match 2).

### 2.2. Analysis

In the visual search task, we recorded participants’ mean reaction times (RTs) and accuracy across various matching conditions and compared attentional capture effects under different experimental conditions using repeated measures ANOVA and paired *t*-tests in SPSS 19.0. Attentional capture effect refers to the difference between RTs in matching conditions and RTs in the non-matching condition (match 0). If there is a significant increase in RTs in matching conditions compared with those in the non-matching condition, it indicates a significant attentional capture effect. For the memory test task, raw memory errors—measured as the degree of difference between the actual and reported colors in CIELAB space—were decomposed into three components of the swap model (Bays et al., 2009; Dube & Al-Aidroos, 2019; Huynh Cong & Kerzel, 2021; Kerzel & Witzel, 2019), following prior studies on working memory resource allocation during visual search (Rajsic & Woodman, 2020); Specifically, we used: (1) a von Mises distribution representing the proportion of responses corresponding to the non-probed item (PSwap), (2) a von Mises distribution representing the precision of responses to the probed item (PSD), and (3) a uniform distribution representing the proportion of random guesses (PGuess). In the theoretical interpretation of these parameters, only PSD was considered to reflect the ongoing distribution of WM resources to the relevant representations (Bays et al., 2009). Thus, in the online Supplemental Materials, we published findings for PGuess and Pswap and concentrated the subsequent analyses on this parameter. The MemToolbox conducted the fits (Ma et al., 2014).

### 2.3. Results

#### 2.3.1. Visual Search Task

Overall, target discrimination accuracy in the visual search task was very high (M > 99%). A seven-way repeated measures ANOVA on accuracy scores for the singleton condition (memory–matching condition: memory 1—baseline; memory 1—match 0; memory 1—match 1; memory 2—baseline; memory 2—match 0; memory 2—match 1; memory 2—match 2) revealed no significant main effect, F(6, 192) = 1.167, *p* = 0.326, η^2^ = 0.035. Thus, accuracy did not vary consistently across conditions.

**Figure 2:**
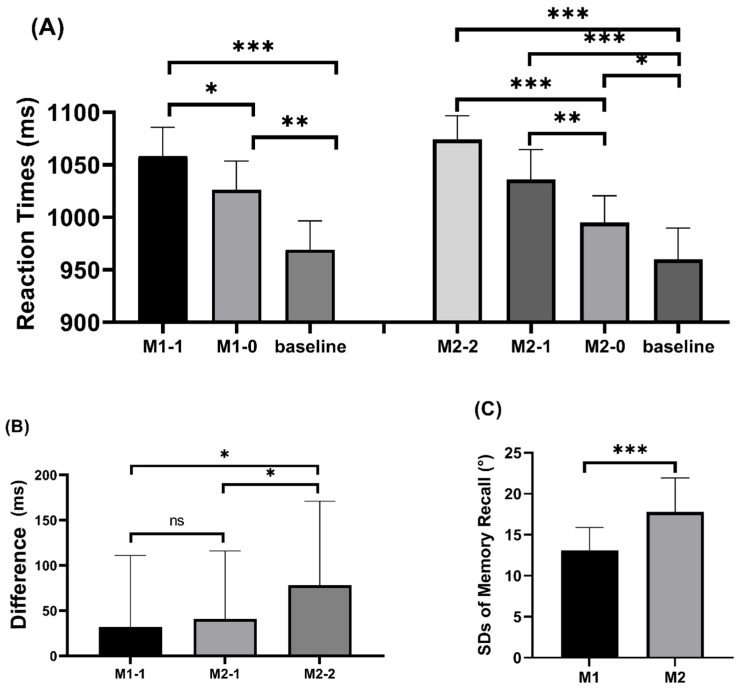
Behavioral results in Experiment 1. Standard deviations (SDs) of memory recall in memory-set-size 1 and memory-set-size 2 conditions; (**A**) Search reaction times in all experimental settings; (**B**) VWM-based attentional capture effects in M1-1, M2-1, and M2-2 conditions; (**C**) Standard deviations (SDs) of memory recall in memory-set-size 1 and memory-set-size 2 conditions. Large SDs signify poor recall, while small SDs suggest precise recall. Standard error is shown by error bars (* *p* < 0.05, ** *p* < 0.01, *** *p* < 0.001, ns not significant).

Reaction times: Figure 2 presents both the standard deviations (SDs) of memory recall and the reaction times for the search task. Only correct trials were included in further analyses, and RTs deviating more than 2.5 standard deviations from a participant’s mean in each condition (fewer than 2% of trials) were excluded. For memory set size 1, a three-way repeated measures ANOVA (matching condition: baseline, match 0, match 1) revealed a significant main effect of the matching condition, F(2, 62) = 16.526, *p* < 0.001, η^2^ = 0.341. Additional pairwise comparisons revealed that there was a significant attentional capture effect under the memory 1—match 1 condition, as RTs in the memory 1—match 1 condition (1058 ms) and memory 1—match 0 condition (1026 ms) were significantly larger than those in the baseline condition (969 ms) (*p* < 0.001, *p* = 0.003), and that RTs in the memory 1—match 1 condition were significantly larger than those in the memory 1—match 0 condition (*p* = 0.028).

For memory set size 2, a four-way repeated measures ANOVA (matching condition: baseline, match 0, match 1, match 2) showed a significant main effect of the matching condition, F(3, 96) = 16.484, *p* < 0.001, η^2^ = 0.34. Follow-up pairwise comparisons revealed a significant attentional capture effect: reaction times in the memory 2—match 1 condition (1036 ms) and the memory 2—match 2 condition (1074 ms) were both significantly longer than in the memory 2—match 0 condition (995 ms) (*p* = 0.004, *p* < 0.001). Additionally, RTs were substantially greater in the memory 2-match 2 condition (1074 ms) than in the memory 2-match 1 condition (1036 ms) (*p* = 0.034). Compared to the baseline condition (960 ms), the memory 2-match 0 condition was noticeably larger (*p* = 0.035).

The WM-based capture effect was calculated as the RT difference between matching and non-matching conditions. A three-way repeated measures ANOVA (memory–matching condition: memory 1—match 1, memory 2—match 1, memory 2—match 2) revealed a significant main effect of the memory-matching condition, F(2, 62) = 4.292, *p* = 0.018, η^2^ = 0.118. Reaction times in the memory 2—match 2 condition (78 ms) were significantly greater than in the memory 2—match 1 condition (41 ms, *p* = 0.034) and the memory 1—match 1 condition (32 ms, *p* = 0.019). No significant difference was found between the memory 1—match 1 condition and the memory 2—match 1 condition (*p* = 0.539). In match 1 condition, there was no significant difference in the attentional capture effect between remembering one item and remembering two. To further evaluate this null difference, we conducted a Bayesian *t*-test using the recommended priors (Rouder et al., 2009) to compare the null model against the alternative. The Bayes factor (BF_01_) of 4.49 provided moderate support for the null hypothesis, indicating that the attentional capture effect was equivalent in the memory 1—match 1 condition and the memory 2—match 1 condition.

#### 2.3.2. Memory Test Task

To evaluate the precision of WM representation, we conducted planned paired *t*-tests on the standard deviations (SDs) of PSD (memory item quantity: 1, 2). The results show that the SDs were smaller in memory 1 condition compared with memory 2 condition (13.08 vs. 17.78), *t*(32) = −6.697, *p* < 0.001. This reveals that the precision of WM representation was higher for remembering one item compared with remembering two items.

### 2.4. Discussion

The effects of WM representation quantity on attentional capture effects and WM resource allocation were examined in Experiment 1. The analysis of the memory test task results reveals that the WM representation accuracy was higher when remembering one item compared with remembering two items. This suggests that a single WM representation under a memory load of 1 obtains more WM resource allocation than a single WM representation under a memory load of 2. The results support a trade-off between the number of VWM representations and their precision, with the precision of each individual representation decreasing as the number of memory items increases (Ye et al., 2017). We report two distinct comparisons. Within the memory-set-size-2 condition, increasing the number of memory-matching distractors increased capture: M2–match 2 produced larger capture than M2–match 1, and both exceeded the non-match baseline. This pattern indicates that multiple memory representations can concurrently bias attention. Across memory loads, holding the match level constant at one (match 1), the capture effect did not differ between memory-set-size 1 and 2. Thus, although the single item in load-1 was remembered with higher precision than each item in load-2, capture magnitude was equivalent for the match1 contrast across loads (Fan et al., 2023; Hollingworth & Beck, 2016; W. Zhang & Luck, 2011; Che et al., 2021; Fan et al., 2019). Furthermore, the attentional capture effect in the search task did not significantly differ between the memory 1—match 1 and memory 2—match 1 conditions. Combining these findings with those of the memory test task, although the resource allocation for a single representation was significantly greater for remembering 1 item compared to remembering 2 items, there was no significant difference in the attentional capture effect between them. The results of Experiment 1 indicate that the allocation of resources for WM representations may not affect attentional capture effects (Huynh Cong & Kerzel, 2022). Together, these results support the view that allocation/precision and capture magnitude can be disassociated: precision was higher at load-1, but capture did not scale with precision when the number of matching distractors was held constant (match 1).

## 3. Experiment 2

In Experiment 1, we measured the allocation of resources for WM representations when remembering 1 or 2 memory items, as well as the amount of attentional capture. The findings from Experiment 1 show that the allocation of resources for a single WM representation varied across different conditions of memory load. However, there was no significant difference in the memory-based attentional capture, suggesting that resource allocation may not affect attentional capture effects. We conducted Experiment 2 to control for the potential impact of varying memory loads on the outcomes of Experiment 1 and to further establish a more rigorous connection between the allocation of WM resources and attentional capture, thus providing evidence that resource allocation in WM representations does not influence attention capture. Following previous research, Experiment 2 involved the presentation of two memory items in the memory array, and the manipulation of resource allocation between the two WM representations was directly implemented using a cueing paradigm (Huynh Cong & Kerzel, 2022), aiming to investigate whether attentional capture is influenced by the allocation of WM resources.

The cueing paradigm employed in this study adopts the retro-cue. In one sense, the allocation of WM resources is flexible, allowing larger resource shares to be given to priority items (Huynh Cong & Kerzel, 2021), and cueing paradigms can be used to manipulate allocation (Huynh Cong & Kerzel, 2022; Souza & Oberauer, 2016). In another sense, the precision of WM representation is jointly determined by preferential encoding during the encoding phase and resource allocation during the maintenance phase (Souza & Oberauer, 2016). The retro-cue paradigm can be used to eliminate the impact of preferential encoding. Building on previous research, Experiment 2 presented two working items and used a retro-cue during the maintenance phase (Landman et al., 2003; Souza & Oberauer, 2016). The cue hinted at which item might be more relevant to the subsequent memory test task, thereby manipulating the allocation of resources between the two WM items (Huynh Cong & Kerzel, 2022; Souza & Oberauer, 2016).

### 3.1. Materials and Methods

#### 3.1.1. Participants

Thirty-seven undergraduate students who were not majoring in psychology were recruited from Hunan University of Science and Technology between the ages of 19 and 22 years (M = 20.08; fourteen males and thirteen females). After the experiment, participants received either RMB 30 (~USD 4.15) or equivalent course credit for their participation. Every subject gave their informed consent and stated that they were right-handed, had normal or corrected-to-normal vision, and were not color blind. The local ethics commission gave its approval to the study.

#### 3.1.2. Stimuli and Procedures

The stimuli and apparatus were identical to Experiment 1, with the following exception. Two memory items, instead of one or two, were presented each time on the memory array in Experiment 2. The delay phase following the memory phase in Experiment 1 was modified to a retro-cue (200 ms) (Landman et al., 2003; Nobre et al., 2007; Souza & Oberauer, 2016). The retro-cues were presented as white arrows centered on the screen. The retro-cue accurately identified the cued item for the next memory task in 75% of trials. Additionally, in the search task, colored squares would only appear either once or not at all. All other procedures remained the same as in Experiment 1.

360 memory trials and 180 search trials (45 trials for each of the four singleton conditions: baseline, non-match, cued-match, and non-cued-match conditions) made up the 540 experimental trials that the participants finished.

#### 3.1.3. Design

This experiment used a single-factor repeated measures design (match condition: baseline, non-match, cued-match, and non-cued-match). The item indicated by the cue was defined as the cued item, while the item not indicated by the cue was defined as the non-cued item. The match conditions were the same as in Experiment 1. In the baseline condition, the targets and distractors in the visual search task were white squares. In the non-match condition, the two memory items and the distractor items in the search task were all different colors. In the cued-match condition, the colored square matched the cued item, whereas in the non-cued-match condition, the colored square matched the non-cued item.

### 3.2. Results

#### 3.2.1. Visual Search Task

Accuracy: Visual search target discrimination accuracy was high (M > 99%). A four-way repeated measures ANOVA (match condition: baseline, non-match, cued-match, non-cued-match) revealed no significant main effect of the memory-matching condition, F(3, 108) = 0.016, *p* = 0.997, η^2^ = 0.001. Thus, accuracy did not vary consistently across conditions.

**Figure 3:**
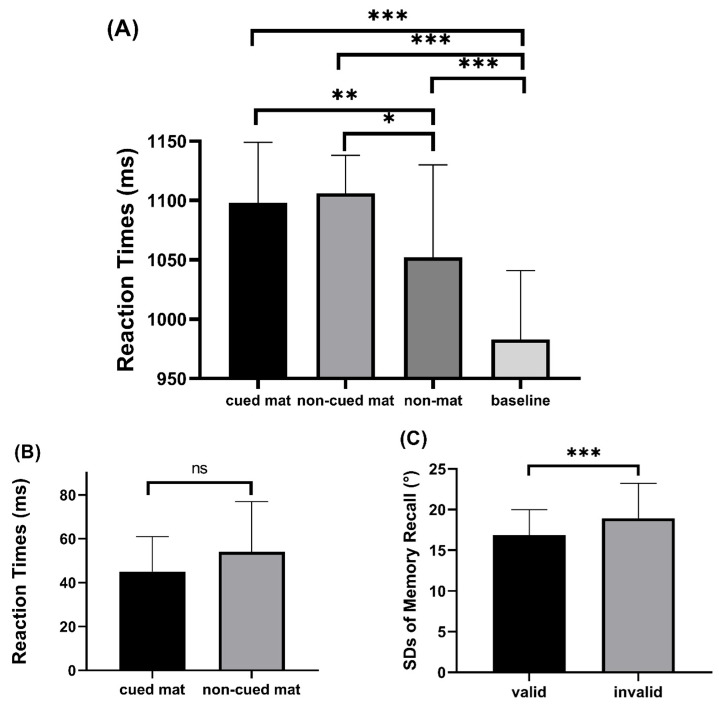
Behavioral results in Experiment 2. (**A**) Search response times across all experimental conditions; (**B**) VWM-based attentional capture effects in cued-match and non-cued-match conditions; (**C**) Memory recall standard deviations (SDs) under situations of valid and invalid retro-cues. Large SDs signify poor recall, whereas small SDs suggest exact recall. Standard error is shown by error bars (* *p* < 0.05, ** *p* < 0.01, *** *p* < 0.001, ns, not significant).

Reaction times: Figure 3 presents both the standard deviations (SDs) of memory recall and the response times for the search task. On average, fewer than 2% of trials were excluded after trimming each participant’s RTs to within 2.5 SDs of the condition mean. A four-way repeated measures ANOVA (match condition: baseline, non-match, cued-match, non-cued-match) on correct-trial search RTs revealed a significant main effect of the match condition, F(3, 108) = 11.312, *p* < 0.001, η^2^ = 0.239. According to post hoc comparisons, the baseline condition (983 ms) was considerably smaller than the cued-match condition (1098 ms), non-cued-match condition (1106 ms), and non-match condition (1052 ms) (*p* < 0.001, *p* < 0.001, *p* = 0.001). There were notable attentional capture effects in both the cued-match and non-cued-match conditions, with the cued-match condition (1098 ms) and non-cued-match condition (1106 ms) being considerably bigger than the non-match condition (1052 ms), *p* = 0.008, *p* = 0.025.

The WM-based capture effect was calculated as the RT difference between matching and non-matching conditions. A planned paired *t*-test compared the attentional capture effects in the cued-match and non-cued-match conditions. The results showed no significant difference between the cued-match condition (45 ms) and the non-cued-match condition (54 ms), t(36) = −0.27, *p* = 0.789. This result suggests that the attentional capture effects in the cued-match and non-cued-match conditions did not differ significantly. To further evaluate this null difference, we conducted a Bayesian *t*-test using the recommended priors (Rouder et al., 2009) to compare the null model with the alternative. The Bayes factor (BF_01_) of 5.467 supported the null hypothesis, indicating that the attentional capture effects in the cued-match and non-cued-match conditions were essentially the same.

#### 3.2.2. Memory Test Task

To evaluate the precision of WM representation, we compared the SDs of PSD on valid retro-cues and invalid retro-cues. The precision of WM representation for items in the valid cue condition was substantially greater than that in the invalid cue condition, as seen by the smaller SDs in the valid cue condition (16.89 vs. 18.94), t(32) = 3.982, *p* < 0.001, as shown by a planned paired *t*-test.

### 3.3. Discussion

In Experiment 2, we presented two WM items and manipulated resource allocation between the two WM representations using retro-cues to investigate the impact of WM resource allocation on attentional capture effects. The results of the memory test task show that the valid cue condition had higher WM precision representation compared to the invalid cue condition. This reveals that the allocation of WM resources to the cued item was greater than that to the non-cued item, supporting the retro-cueing effect (Souza & Oberauer, 2016). The results of the visual search task showed significant attentional capture effects in both the cued-match and non-cued-match conditions; however, there was no significant difference between them. The findings of the experiment are consistent with previous studies using similar experimental paradigms (Che et al., 2021; Dube & Al-Aidroos, 2019; B. Zhang et al., 2018). The results of Experiment 2 further confirm the conclusion of Experiment 1, indicating that the allocation of WM resources does not significantly affect the magnitude of attentional capture effects.

## 4. General Discussion

This study adopted the attentional capture paradigm and continuous delayed-estimation tasks to explore the relationship between the allocation of WM representations and attentional capture. In Experiment 1, we manipulated WM resources by presenting one or two memory items, measured attentional capture effects and the allocation of WM resources, and explored the relationship between them. In Experiment 2, we presented two memory items and then presented retro cues during the memory maintenance stage to change the allocation of WM resources between the two representations. We assessed whether the attention capture effect was impacted and looked more closely at the connection between WM resource allocation and attention capture effects. The findings demonstrate that in the visual search task, items with high WM precision—those that dedicated more WM resources—did not produce stronger attention capture effects. This result implies that the amount of attentional capture effects of WM representations is independent of WM resource allocation. Although our experiments used abstract, color-defined stimuli to isolate the mechanism, the same working-memory–guided selection processes plausibly operate in everyday tasks. In driving, multiple concurrently active templates (e.g., brake lights, lane markings, destination signage) could bias attention toward any matching cue, consistent with our finding that capture scales with the number of matches while not tracking representational precision. In reading, top-down templates for upcoming lexical or spatial targets can guide fixation selection without necessarily altering search efficiency, paralleling our dissociation between capture magnitude and efficiency. In multi-object monitoring (dashboards and control rooms), maintaining several candidate targets should prioritize any item that matches an active template, even when individual representations are noisier. We note limits on generalization (static, color-diagnostic displays; restricted semantics) and outline that future work should use dynamic, context-rich scenes and eye-tracking to test whether the template-count versus precision relationship governs real-world orienting.

### 4.1. Multiple WM Representations Capture Attention Simultaneously

In this study, we employed the attentional capture paradigm; the gap-location task, which has lower search efficiency for the search task; and continuous delayed-estimation tasks, which need more precision for the memory test task. In continuous delayed-estimation tasks, subjects were required to accurately remember the colors of memory items and then recall and select the corresponding colors in the following estimation tasks. The continuous delayed-estimation tasks we employed necessitated greater precision in WM representation and consumed a larger amount of WM resources, as compared to the change detection task, thus using more WM (W. Zhang & Luck, 2011). This adjustment could effectively lower the cognitive resources of cognitive control, diminish the inhibition of distractor items on the search display (Che et al., 2021) and evaluate attentional capture effects steadily.

Experiment 1 showed that the RT observed in both the memory 2-match 2 and memory 2-match 1 conditions was significantly elevated compared with that observed in the match 0 condition, indicating a significant attentional capture effect. Additionally, compared to memory 2-match 1 condition, the attentional capture effect was stronger in memory 2-match 2 condition. This finding suggests that multiple WM representations can simultaneously capture attention. Our findings align with previous research (Fan et al., 2023; Hollingworth & Beck, 2016; Che et al., 2021; Fan et al., 2019) supporting the MIT hypothesis (Beck et al., 2012), which suggests that multiple representations can simultaneously guide visual search. During the visual search task conducted in Experiment 2, both the cued-match and non-cued-match conditions exhibited RTs that were longer than those observed in the non-match condition, leading to a significant attentional capture effect. Notably, no significant difference was observed in the magnitude of attentional capture effects between these two conditions. This finding aligns with previous studies that have employed similar experimental paradigms (Che et al., 2021; B. Zhang et al., 2018). By combining the results of both experiments, we posit that multiple WM representations possess the capacity to capture attention, thereby supporting the MIT hypothesis.

### 4.2. The Allocation of WM Resources Does Not Affect the Attention Capture Effect

The findings from Experiment 1 show that in the memory test task, remembering a single item resulted in reduced PSD and enhanced precision of WM representation compared with remembering two items. These results suggest that as the number of memorized items increases, the individual representations for each item become less precise (Xu et al., 2018). Previous research has found that precision representation reflects the allocation of WM resources (Huynh Cong & Kerzel, 2021). Our results indicate a greater allocation of WM resources for remembering one item compared with remembering two items. In the search task, there were no significant differences in the magnitude of RTs between the Match 1 and non-match conditions when remembering one item or two items, indicating no significant difference in the magnitude of attentional capture effects. This finding is consistent with results from multiple studies using change detection procedures (Fan et al., 2023; Hollingworth & Beck, 2016; Che et al., 2021; Fan et al., 2019). Our findings indicate that the allocation of WM resources is higher when remembering a single item compared to when remembering two items. However, the subsequent visual search task revealed no significant difference in the magnitude of attentional capture effects between the two conditions, thereby supporting the notion that attentional capture effects do not necessarily correspond to WM resource allocation. Based on the aforementioned findings, we deduce that the allocation of resources toward WM representations may not exert an influence on attentional capture effects. This implies that determining the extent of memory-based attentional guidance may need more than just resource allocation (Fan et al., 2023).

Previous research has found that WM resources can be flexibly allocated between representations based on the relevance of subsequent tasks (Bays & Husain, 2008) and that the retro-cues used during the maintenance phase, Experiment 2, can control how WM resources are distributed across representations (Huynh Cong & Kerzel, 2022; Souza & Oberauer, 2016). The results revealed that in the memory probe task, the WM representation precision of the cued item was higher than that of the non-cued item. This reveals that the cued item received a higher allocation of WM resources than the non-cued item, supporting the retro-cueing effect (Souza & Oberauer, 2016). In the search task, when examining the differences in attentional capture effects of distractor items, there were no significant differences observed between the cued-match and non-cued-match conditions. This result aligns with previous research (Che et al., 2021; B. Zhang et al., 2018), showing that variations in the distribution of WM representation resources do not influence the magnitude of attentional capture during visual search. It also reinforces the findings from Experiment 1 (Fan et al., 2023), which suggest that the extent of attentional capture cannot be determined solely by the allocation of WM resources.

### 4.3. Attentional Capture and the Stages of Attentional Guidance

Previous research on the influence of the allocation of WM resources on attentional guidance has produced inconsistent findings, and the underlying mechanisms remain unclear. While a study utilizing the dual target search paradigm has found that the allocation of WM resources can enhance visual search efficiency (Huynh Cong & Kerzel, 2022), studies utilizing attentional capture paradigms—including this study and numerous previous investigations (Fan et al., 2023; Hollingworth & Beck, 2016; Che et al., 2021; Fan et al., 2019)—have revealed that the allocation of WM resources is insufficient to influence attentional capture. The potential explanation for this phenomenon may reside in the divergent outcomes yielded by distinct experimental paradigms, specifically pertaining to the disparate stages of attentional guidance investigated through various experimental tasks. For example, Ort proposed that memory-based attentional guidance involves three processes (Ort & Olivers, 2020): The first step, **preparation**, involves storing a representation that can bias incoming sensory input to prioritize visual objects that match it. The second step, **selection**, uses this representation to guide attention toward matching information in the visual field. The third step, **postselection processing**, is where the observer verifies that the chosen object is indeed the target. In the dual-target search paradigm, WM representations act as the targets for the search task and play a central role in the memory-based attentional guidance process (Huynh Cong & Kerzel, 2022; Ort et al., 2019; Ort & Olivers, 2020). In contrast, in the attentional capture paradigm, the occurrence of attentional capture effects involves two stages: an early stage of memory-driven automatic attentional capture and a later stage of cognitive control-based inhibition of attention. The memory matching stimulus is suppressed by cognitive control, subsequent to which attention is automatically captured by it during the early stage of attentional guidance; thus, the attentional capture effect does not fully reflect the entirety of the visual search process (Hu & Zhang, 2016). Our results indicate that the allocation of WM resources does not affect attentional capture effects, which may be related to specific processes in visual search.

Subsequent research has explored the mechanisms underlying different stages of visual search. Several studies have distinguished between the costs associated with template preparation and template engagement during selection. They found that preparing for selecting two objects was associated with only small neural and behavioral costs, whereas selecting multiple targets simultaneously was associated with higher costs (Ort et al., 2019). This implies that the capability of the top-down bias preparation process is greater than that of the process of applying those biases to choose relevant data from the visual input (Ort et al., 2019; Ort & Olivers, 2020). Because the storage of attentional templates in VWM is widely acknowledged (Carlisle & Woodman, 2011; Desimone & Duncan, 1995; Duncan & Humphreys, 1989; Olivers et al., 2011), coupled with the recognition of WM as a limited resource (Bays et al., 2009; Bays & Husain, 2008; Huynh Cong & Kerzel, 2022; Ma et al., 2014), we deduce that the reduced capacity limitation observed during the preparatory stage of visual search can be likened to a diminished constraint on WM resources. Our study’s finding that the allocation of WM resources is insufficient to exert influence on attentional capture effects may be attributed to the fact that attentional capture effects predominantly reflect the preparation stage of memory-based attentional guidance. However, to confirm this result, further research is needed in the future.

## 5. Conclusions and Limitations

Across two experiments, we demonstrated the intricate relationship between the allocation of WM resources and attentional capture effects. By seamlessly integrating the attentional capture paradigm with continuous delayed-estimation tasks and retro-cue tasks, we aimed to gain a deeper understanding of this association. The findings of this investigation reveal that when WM resources were allocated toward a single representation of one item, it surpassed the allocation for each representation of two items. Nevertheless, the magnitude of attentional capture effects remained equivalent across both scenarios. Furthermore, our results show that the allocation of WM resources toward cued items exceeded that of non-cued items; however, the attentional capture effects remained unchanged. These findings suggest that within the attentional capture paradigm, the distribution of WM representation resources does not exert a significant influence on the magnitude of memory-based attentional capture effects. To aid interpretation of the null effects, we evaluated sensitivity and potential range restrictions. Based on the observed within-participant variability in the capture score (RT_{match} − RT_{non-match}) and the correlation between matched and non-matched RTs, the design provided adequate power (α = 0.05; 1−β = 0.80) to detect small effects of practical interest; any true differences smaller than this minimal detectable difference were negligible for our purposes. Complementary equivalence tests (TOSTs) using a pre-specified smallest effect size of interest (SESOI) indicated that the critical contrasts were statistically equivalent to zero within that margin, and Bayesian analyses yielded Bayes factors favoring the null over alternatives of typical magnitude in this literature. Importantly, the manipulations were effective: load and retro-cue factors robustly altered working-memory precision, and within load-2, capture was larger with two matching distractors than with one, confirming that the task elicits measurable guidance when present. Distributional diagnostics of reaction times (medians, interquartile ranges, and 5th–95th percentiles) showed ample dynamic range without evidence of ceiling or floor effects, and accuracy ceilings did not constrain RT-based capture measures, which were computed on correct trials. Taken together, these considerations suggest that the absence of differences in the targeted contrasts reflects genuinely small or absent effects rather than insufficient power or range limitations.

However, it is important to take into account that this study still has certain limitations. First, behavioral research serves as the foundation for the evidence supporting this study’s conclusions. Nevertheless, no research was conducted in the area of electrophysiology, and behavioral assessments might not be very sensitive, which could mean that minute variations did not reach significant levels. Further investigations on the relationship between WM resources and attentional capture should make use of techniques like brain imaging and electrophysiology. Second, rather than focusing on the intricacies and underlying mechanisms of attentional capture, this study examined the relationship between WM resource allocation and attentional capture. Future investigations into the intrinsic mechanisms of attentional capture are necessary to shed light on the reasons behind the discrepancies in the findings of memory-guided attention studies conducted under various paradigms.

## Data Availability

Data supporting the results may be shared by the authors upon reasonable request.

## Supplementary Materials

The following supporting information can be downloaded at: https://www.mdpi.com/article/10.3390/bs16010104/s1, Table S1. Experiments 1–2—Mean RTs (ms) and capture scores.
