# Supplementary material for: Identical Attentional Capture with Different Working Memory Representation Precision
Source: Behav Sci (Basel). 2026 Jan 13;16(1):104. doi: 10.3390/bs16010104 (PMC12837926; doi:10.3390/bs16010104)
Supplement: Supplementary file 1 [file behavsci-16-00104-s001.zip › behavsci-3866115-supplementary.pdf]

Table S1. Experiments 1–2 — Mean RTs (ms) and capture scores

Experiment	Memory load	Condition	Mean RT (ms)	Capture vs non-match (ms)
1	1 item	Baseline	969	—
1	1 item	Non-match (match 0)	1026	—
1	1 item	Match 1	1058	32
1	2 items	Baseline	960	—
1	2 items	Non-match (match 0)	995	—
1	2 items	Match 1	1036	41
1	2 items	Match 2	1074	78
2	—	Baseline	983	—
2	—	Non-match	1052	—
2	—	Cued-match	1098	45
2	—	Non-cued-match	1106	54
